# Complete chloroplast genome sequence of *Fagus hayatae* Palib. (Fagaceae)

**DOI:** 10.1080/23802359.2022.2080015

**Published:** 2022-06-02

**Authors:** Chaoyang Jiang, Wenqian Fan, Lijun Chen, Xiaohong Gan

**Affiliations:** a College of Life Sciences, China West Normal University, Nanchong, China; b Key Laboratory of Southwest China Wildlife Resources Conservation (Ministry of Education), China West Normal University, Nanchong, China

**Keywords:** *Fagus hayatae*, chloroplast genome (cpDNA), phylogenetic position

## Abstract

Complete chloroplast genome (cpDNA) sequence of *Fagus hayatae* Palib. is yet to be reported, and the phylogenetic position of this species is still under debate. In this study, the complete cpDNA sequence of *F. hayatae* was determined from Illumina NovaSeq pair-end sequencing data. Results revealed that it has a sequence length of 158,360 bp and contains 131 annotated genes, which consist of 83 protein-coding genes, 40 tRNA genes, and eight rRNA genes. The phylogenetic analysis of the complete cpDNA sequence indicates that *Fagus* represents a monophyletic clade within Fagaceae. The species relatedness between *F. hayatae* and *F. engleriana* is relatively close.

First discovered by Palib in 1911 (https://www.tropicos.org/name/Search?name=fagus), *Fagus hayatae* Palib (*F. hayatae*) is a China-endemic species belonging to the family Fagaceae (Huang et al. 2017). It is of great interest to researchers exploring plant floristic differences and evolution (Zhang 2017). Although the complete chloroplast genome (cpDNA) sequences of other *Fagus* species, namely, *F. sylvatica*, *F. japonica*, *F. crenata*, and *F. engleriana*, have been reported, the cpDNA sequence of *F. hayatae* is yet to be reported (Jong-Soo et al. 2019; Worth et al. 2019; Yang et al. 2020; Ulaszewski et al. 2021). Furthermore, the phylogenetic position of *Fagus* remains unclear. Li (1996) indicated that *F. hayatae* is close to *F. lucida* and far from *F. engleriana* in terms of relatedness. However, Kato et al. (2000) believed that the kinship between *F. hayatae* in Taiwan and *F. crenata* in Japan is the closest. In this study, we reported the complete cpDNA sequence of *F. hayatae* and explored its phylogenetic position in Fagaceae through phylogenetic analysis.

The plant materials of *F. hayatae* were collected from Micang Mountain Nature Reserve, Sichuan, China (E 106°33′47.31″ and N 47.31°39′71.37″). The voucher material was retained at the herbarium of College of Life Sciences, China West Normal University (voucher number: MCS202005). Whole genomic DNA was isolated from dehydrated leaves by the CTAB method (Doyle and Doyle 1987). Total DNA was detected using a Quant-iT PicoGreen dsDNA assay kit, and DNA integrity was detected by 1% agarose gel electrophoresis. The extracted cpDNA was sequenced with NovaSeq Control Software V1.7.0, the DNA library was constructed by Illumina TruSeq DNA sample preparation guide, and the template size was 400 bp. The annotation of *F. sylvatica* (MK598696) was introduced as a seed input to assemble the cpDNA of *F. hayatae* by NovoPlasty (Dierckxsens et al. 2016). A general time-reversible model was selected as the best nucleotide substitution model to construct the phylogenetic tree, and the bootstrap value was calculated from 1000 replicate analyses. Evolutionary history was inferred using the maximum-likelihood method in MAGA7.0 (Kumar et al. 2016). Then, the multiple sequence alignment of the complete cpDNA of 15 species from Fagaceae, including *F. hayatae*, was analyzed by MAFFT (Katoh and Standley 2013). *Quercus*, *Lithocarpus*, *Castanea*, and *Castanopsis* were selected as the outer groups for establishing a phylogenetic tree (Figure 1). A guide value (%) was displayed above the branch (Mader et al. 2019).

**Figure 1.**
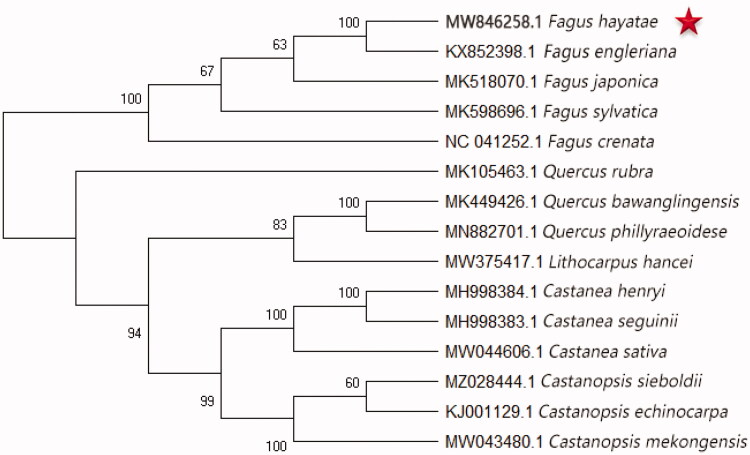
Numbers on the nodes are bootstrap values from 1000 replicates. A phylogenetic tree of 15 species belonging to Fagaceae was constructed on the basis of the chloroplast genome sequence. *Quercus*, *Lithocarpus*, *Castanea*, and *Castanopsis* are selected as the outgroup.

A typical quadripartite structure has 158,360 bp (GC content: 36.85%) and is composed of a large single-copy region (87,661 bp), a small single-copy (18,892 bp), and a pair of inverted repeat regions (25,903 bp). A total of 131 *annotated genes*, including 83 protein-coding genes, 40 tRNA genes, and eight rRNA genes, were obtained. The smallest genetic distance was observed between *F. hayatae* and *F. engleriana*, suggesting that their phylogenetic relationship was the closest (Figure 1). Moreover, the cpDNA sequences of five available *Fagus* species formed a cluster. Consistent with previous results (Mader et al. 2019), our findings indicated that *Fagus* formed a monophyletic clade in Fagaceae.

## Data Availability

The genome sequence data supporting the findings of this study are openly available in GenBank of NCBI at https://www.ncbi.nlm.nih.gov/nuccore/MW846258 under the accession number MW846258.1. The associated BioProject, BioSample, and SRA numbers are PRJNA718652, SAMN18558391, and SRR14116373, respectively.
